# Large-Scale Crustal-Block-Extrusion During Late Alpine Collision

**DOI:** 10.1038/s41598-017-00440-0

**Published:** 2017-03-24

**Authors:** Marco Herwegh, Alfons Berger, Roland Baumberger, Philip Wehrens, Edi Kissling

**Affiliations:** 1 Institute of Geological Sciences, University of Bern, Baltzerstrasse 1+3, CH-3012 Bern, Switzerland; 2 Now at Federal Office of Topography, Swiss Geological Survey, Seftigenstrasse 264, 3084 Wabern, Switzerland; 3 Institute of Geophysics, ETH Zürich, Sonneggstrasse 5, CH-8092 Zürich, Switzerland

## Abstract

The crustal-scale geometry of the European Alps has been explained by a classical subduction-scenario comprising thrust-and-fold-related compressional wedge tectonics and isostatic rebound. However, massive blocks of crystalline basement (External Crystalline Massifs) vertically disrupt the upper-crustal wedge. In the case of the Aar massif, top basement vertically rises for >12 km and peak metamorphic temperatures increase along an orogen-perpendicular direction from 250 °C–450 °C over horizontal distances of only <15 km (Innertkirchen-Grimselpass), suggesting exhumation of midcrustal rocks with increasing uplift component along steep vertical shear zones. Here we demonstrate that delamination of European lower crust during lithosphere mantle rollback migrates northward in time. Simultaneously, the Aar massif as giant upper crustal block extrudes by buoyancy forces, while substantial volumes of lower crust accumulate underneath. Buoyancy-driven deformation generates dense networks of steep reverse faults as major structures interconnected by secondary branches with normal fault component, dissecting the entire crust up to the surface. Owing to rollback fading, the component of vertical motion reduces and is replaced by a late stage of orogenic compression as manifest by north-directed thrusting. Buoyancy-driven vertical tectonics and modest late shortening, combined with surface erosion, result in typical topographic and metamorphic gradients, which might represent general indicators for final stages of continent-continent collisions.

## Introduction

Convergent plate motions may ultimately result in the collision between two continents with the closure of an oceanic basin. Deformation after the initial welding of the two continents is considered collisional and this sequence is incorporated into the classical subduction zone model. Within this framework, young fold-and-thrust belts at the fronts of such collisional orogens are commonly interpreted to represent a final compressional stage (e.g. Appalachians; Jura mountains). With the onset of collision, the slab-pull forces driven by the dense subducting oceanic lithosphere start to become counterbalanced by the buoyancy of the lower density continental crust, as it is pulled into the subduction channel. The result being that the subducting plate motion and plate convergence rates decrease. As a consequence of strong shear forces between the buoyant continental and dense oceanic lithosphere of the lower plate, a subducting slab may eventually tear and break off owing to complex thermo-mechanical feedback mechanisms in the lithospheric mantle^1^. A reduction in slab-pull after a slab breakoff enhances the buoyancy component of the remaining lithosphere, causing the orogen to rise^2^. In addition, subducting slabs can also retreat^3^, by a process referred to as slab rollback. Modern geophysical techniques like seismic tomography provide deep insights into the upper mantle proofing the existence of subducting slabs, slab breakoffs and slab rollback^4^.

In the case of the Alps, which are considered as a classical compressional orogen, seismic tomography was recently used to hypothesize a rollback mechanism in the subducting European lithosphere as major orogenic process^2^. It is suggested that delamination^5^ and accretion of the subducting plate during rollback induce crustal thickening. This increases the surface topography promoting erosion and subsequent sedimentation in the Molasse foreland basin^2^. Such a scenario requires accommodation by severe pervasive vertical movements and prominent deformation structures within the orogen. Despite restricted domains documenting vertical displacements (e.g. Southern Steep Belt^6, 7^), yet no observations exist documenting and expressing a pervasive dominantly subvertical tectonic deformation style in the External Alps.

In this study, we shed new light on the aforementioned hypothesis and unravel the structural link between post-collision convergence and rollback-related deformation. For this purpose, we study the Aar massif (Swiss Alps), the largest of the External Crystalline Massifs (EMC), and utilize it to document the young and severe vertical uplift^8^ of an entire large-scale crustal block.

## Tectonic architecture: the past view

Because of their crustal dimensions and their arcuate alignment along the N to SW front of the Alpine chain, the EMC (Supplementary Fig. 1) have been recognized for a long time as major structures of the Alpine orogen^7–13^. The ECM are characterized as exhumed middle to upper crustal blocks, expressed by lentoid shapes in map view (25–60 km × 10–25 km). Assuming an average upper and middle continental crustal thickness of 20 km^14^, ECM therefore represent significant individual crustal volumes of up to 30’000 km^3^. The ECM mainly consist of pre-Alpine polymetamorphic gneisses, Late Palaeozoic sediments and volcanic rocks as well as post-Variscan granitoids overlain by a Mesozoic to Cenozoic sedimentary cover.

The available geodynamic models are inspired by a traditional continent–continent collision concept applied to the Adriatic and European plates, including partial subduction of the Alpine foreland and rotation and indentation of the Adriatic plate^15^. The occurrence of the ECM has been related to progressive continent-continent collision with piling of nappe stacks as well as indentation of Adria at lower crustal levels^12, 15^. It is postulated that in a late stage of the Alpine orogeny major out-of-sequence thrusts evolved, along which the ECMs were bulged, tilted and exhumed. In the case of the Aar massif three major kinematic models were proposed for this evolution: (i) an antiformal nappe stack of upper crustal rocks^16^; (ii) crustal-scale buckling^16^ or (iii) formation of a pop-up structure because of north- and south-directed thrusts at both ends of the massif, respectively^16, 17^. Additionally, a substantial NW-SW shortening with an associated flattening is documented for the central part of the Aar massif^17, 18^. Mass balance considerations^16, 19^ require a basal detachment in the middle crust as common ground of all three models, above which the compression-induced crustal thickening imposes the up-doming of the crystalline basement (see Fig. 5 in ref. 16). Although being kinematically reasonable, two severe problems arise with aforementioned models: Missing evidence for massif internal thrusts (model i) or backthrusts (model iii) accommodating considerable displacements and peak metamorphic temperatures that are too low (250–450 °C) to allow for a pervasive ductile folding of the crystalline basement rocks (model ii). Realizing these problems, in the following we discuss an alternative geodynamic setting, which allows exhumation of such a large-scale crustal basement block.

## Tectonic architecture: new aspects

**Figure 1:**
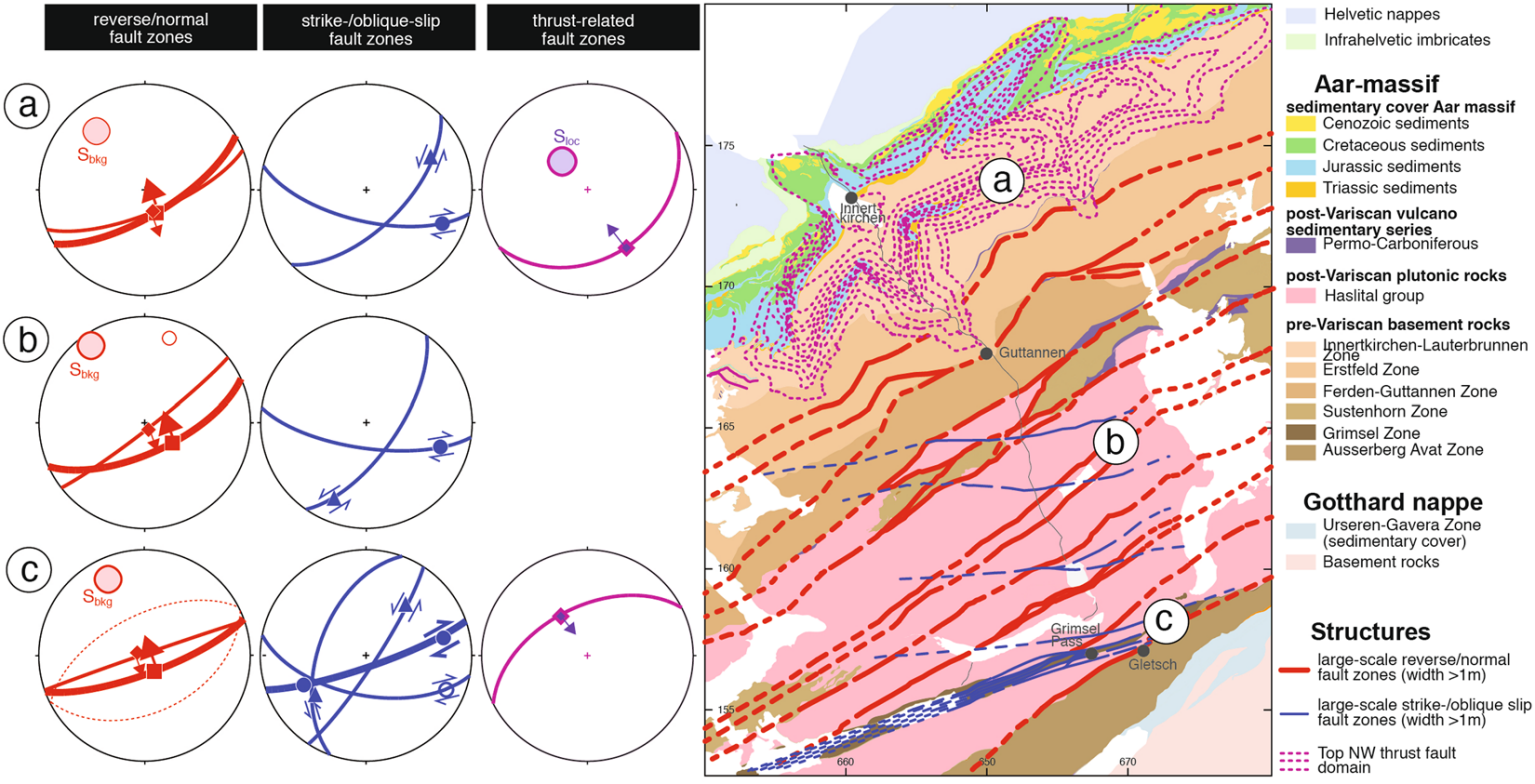
Structural data. Right side: Map of the Central Aar massif with the major geological units and fault zones with widths >1 m. Left side: Stereo plots with great circles of mean planes of reverse/normal, strike-/oblique slip and thrust faults, associated stretching lineations with shear sense or transport direction of the hanging wall block as well as poles of the background strain foliation and the local new foliation of the thrust domain in the north. Full data set given in Supplementary Fig. 4. The map was created with Adobe Illustrator 5.5 (https://helpx.adobe.com/illustrator.html).

**Figure 2:**
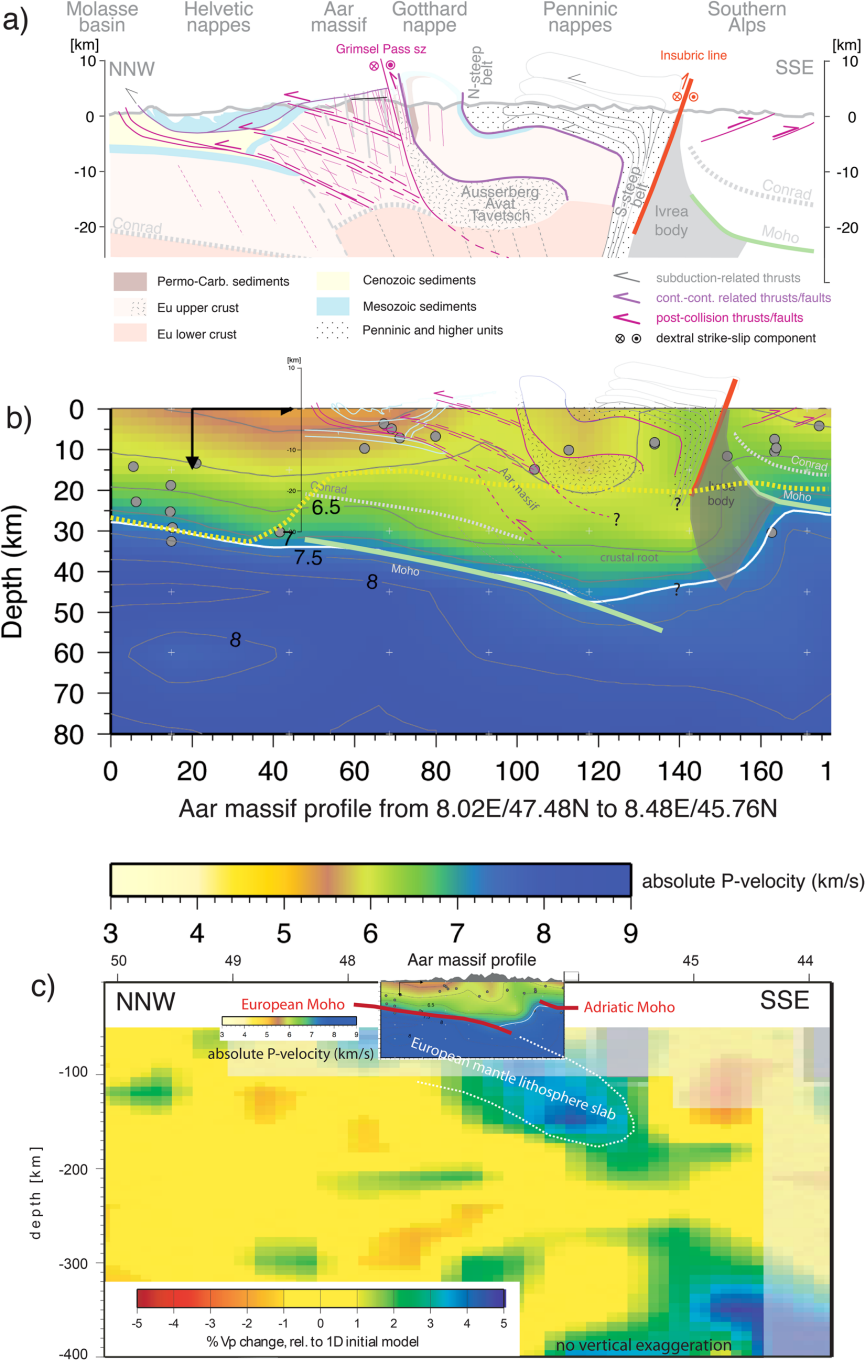
Crustal-scale section from the Molasse basin to the Southern Alps. For profile trace see Supplementary Fig. 1. (**a**) Constructed tectonic section based on previous knowledge of the Helvetics^28^, the Penninics^31^ and the Southern Alps^33^. Note the exhumation of the large-scale crustal block of the Aar massif (i) first along the steep south dipping shear zones with south block up movements and (ii) later by NW-directed thrusting along moderately south dipping shear domains. (**b**) Seismic p-wave tomography^23^ along the same profile trace, showing a strong accumulation of lower crust (Vp 6–6.5 km/s) underneath the Aar massif and the deep deflection of upper crust (Vp 5–5.5 km/s) below the Gotthard nappe. Yellow stippled line shows the lower limit of the seismically active zone. Thin red and white lines represent outlines of thrusts/faults and lithological boundaries delineated in (**a**). Black arrows: depth and horizontal dimensions of local tomography cells; white crosses represent centers of these cells and the grey circles the earthquake hypocenters used for the calculations. Moho location based on local tomography^45^. (**c**) Teleseismic tomography image^4^ with the studied section showing (**b**) as inset. Note remnant of the European slab sitting underneath the Alps.

Despite numerous thematic and local structural studies, surprisingly little is so far known about the large-scale internal structure of the Central Aar massif and its massif-wide kinematic evolution. We use a combination of kinematic analyses^20^, 3D modeling of large-scale fault zones^21^, geophysical modeling of the Central Alpine region^22^ and data from recent high-resolution local earthquake tomography^14, 23^, to construct a crustal-scale tectonic NNE-SSW section ranging from the Molasse basin to the Southern Alps (Figs 1 and 2, Supplementary Fig. 1).

Teleseismic tomography reveals a cold mantle lithosphere slab of the European plate^4^, which is south dipping underneath the Adriatic plate down to depths of about 180 km (Fig. 2c). The high-resolution crustal tomography (Fig. 2b) presents a 35 km thick Adriatic continental crust with its Moho at its northern end being offset by a vertical jump of ~25 km down to the subducted European Moho. In this section, the crustal root of the Alps reaches maximum depths of about 50 km (Fig. 2b) consisting mainly of lower crustal rocks (P-wave velocity (Vp) ~6.5 km/s)^22, 24^. The manifestation of an upward deflection of the Vp 6.0 km/s contour line also shows that lower and middle crustal material has accumulated beneath the Aar massif. On the contrary, the downward deflection south of the Aar massif corresponds to an accumulation of upper crustal rocks (Vp < 6.0 km/s) being situated on top of the still thickened lower crust.

In terms of mid to upper crustal structures, refraction and reflection seismics^25, 26^ and surface information^27, 28^ established a well-known structure for the northern part of the section. Here former European passive continental margin, its Mesozoic sedimentary cover as well as the inverted part of the Swiss Molasse basin are south dipping with the N-directed thrusts of the Helvetic nappes on their top (Fig. 2a, left part). In combination with uplift of mantle rocks of the Ivrea body, the overlaying lower and middle crust of the former Adriatic passive continental margin was bent up, and S-vergent thrusting dominated the post-collisional history^29^. The Insubric Line as a major strike-slip and backthrust of the Alps defines the northern termination of the Southern Alps, separating their low-grade metamorphic rocks from amphibolite grade metamorphic rocks of the Penninic nappe stack (Fig. 2a, right part). This nappe stack is subvertically reoriented (Southern Steep Belt) near the Insubric Line and rotates into subhorizontal position towards the North (late stage backfolding^30, 31^). The Penninic nappe stack can be seen in a very simplified view as the suture zone between the European and Adriatic plates (including the Briançon terrane), comprising in parts remnants of the former subducted oceanic crust/exhumed mantle^32^, as well as extended European continental crust. The lower plate rocks (formerly ocean and parts of the European plate) were accreted to the Adriatic upper plate (tectonically accreted lower plate (TALP)^33^).

**Figure 3:**
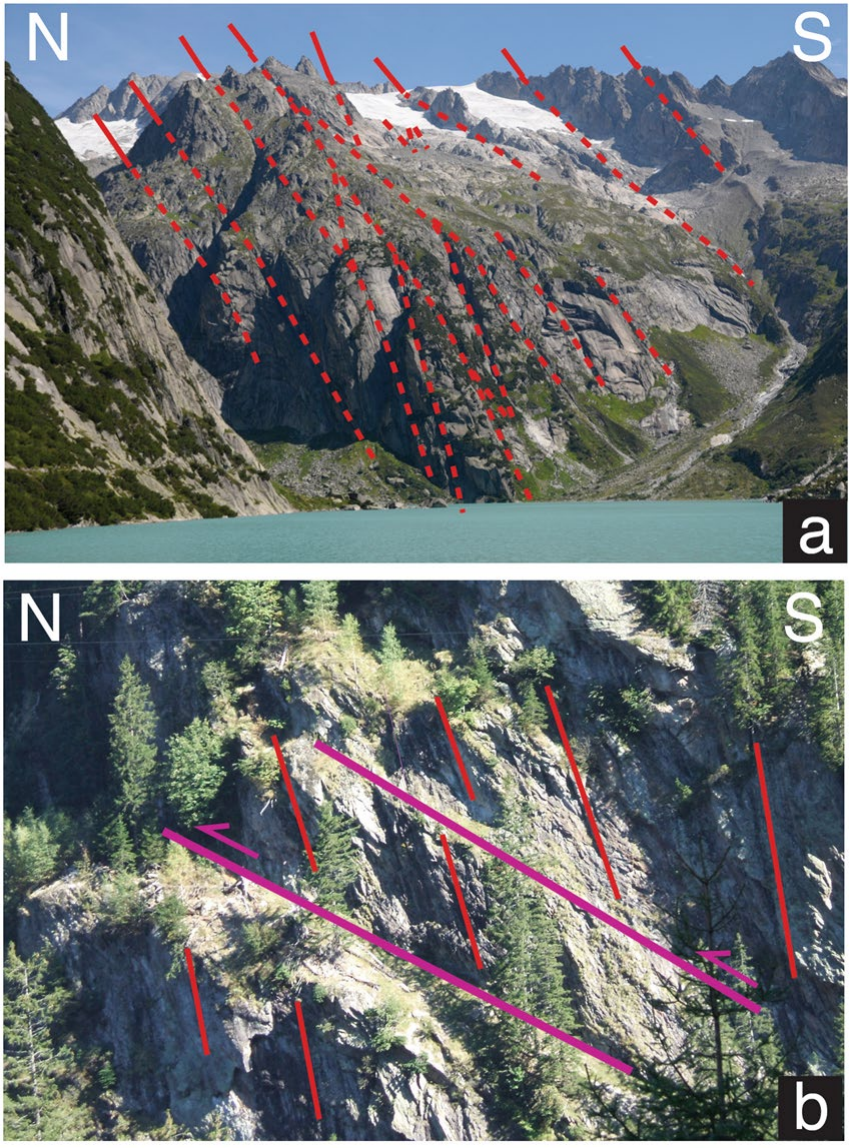
Field photographs from the (**a**) S- and (**b**) N-Aar massif. (**a**) Granitoid heavily dissected by a network of steeply S-dipping Alpine shear zones (swiss coordinates 669’000/163’725); (**b**) polymetamorphic gneisses with old steeply S-dipping Alpine shear zones being cut by flat reverse faults (swiss coordinates 667’901, 174’355).

**Figure 4:**
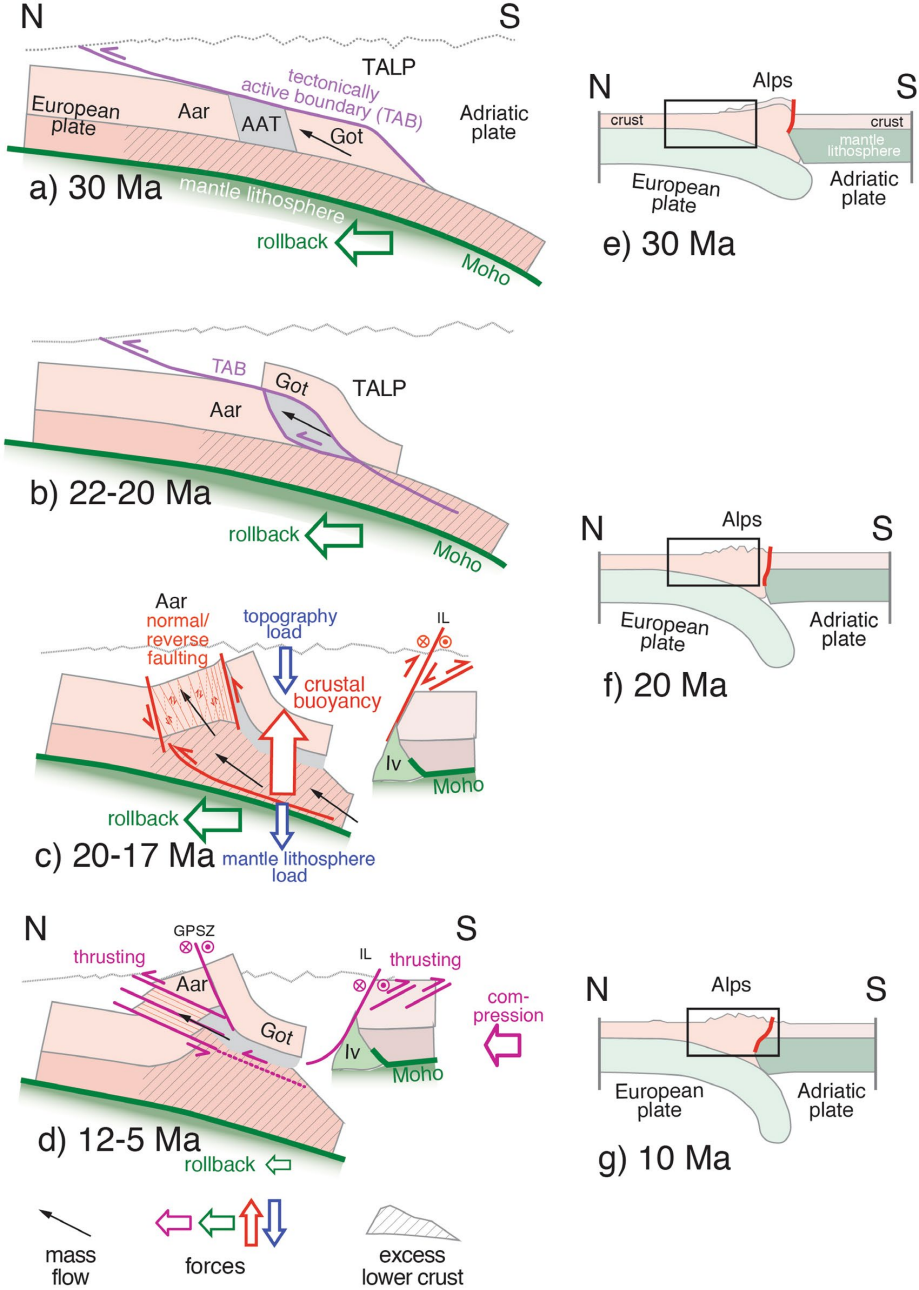
Schematic evolution of the Aar-massif and related forces. (**a**) Rollback of the subducted European plate underneath the accretionary wedge of the Adriatic plate, onset of emplacement of Gotthard (Got) nappe. (**b**) Onset of basal accretion of the Ausserberg-Avat-Tavetsch zone (AAT). (**c**) Delamination of lower crust (i) reducing lithospheric mantle load, which (ii) promotes the subvertical buoyancy driven uplift of the entire crustal segment of the Aar massif (Aar). Note crustal thickening and the steep reverse/normal faulting. (**d**) Ceasing of rollback effect and final N-directed thrusting in the Aar massif by remaining compressional forces. (**e**,**f**) Lithosphere-scale overview of the evolution of the Alps after^2^. Insets represent domains of sketches of the left column. Iv: Ivrea mantle body, IL: Insubric line, GPSZ: Grimsel Pass shear zone.

It is well known from surface information that the amphibolite grade asymmetric late stage fold of the Gotthard nappe is situated N of the Penninic nappe stack (Fig. 2a, Northern Steep Belt^6, 7^). The Gotthard nappe is separated by a zone of polymetamorphic basement units (Ausserberg-Avat Zone of the Aar massif and Tavetsch-nappe, in the following summarized as “AAT”) from the large crystalline block of the Aar massif to the N. By iteratively combining field information (e.g. Figs 1, 2 and 3), line- and mass-balanced tectonic restoration as well as information from seismic tomography (Fig. 2b), the crustal structure of this domain has been reconstructed (Fig. 2a). The Gotthard nappe follows the shape of Penninic nappe stack representing the low Vp domains in Fig. 2b (see also refs 15, 31 and 33). The remaining low velocity domain underneath requires the presence of additional upper to mid-crustal rocks, which we present by the underthrusted AAT. In this way, these polymetamorphic gneisses and Late Paleozoic sediments actually present the basement of the Helvetic nappes, which were sheared off from their substratum prior to the emplacement of the Gotthard nappe (Fig. 4).

In accordance with previous studies^18, 20, 21^, we suggest a crustal thickening in the Aar massif, which increases towards S (Figs 2a and 4). This interpretation is in line with the Vp structure of seismic tomography (Fig. 2b). A degree of uncertainty exists in the location of the Conrad discontinuity, which is not directly geophysically resolved, but is constrained by area balancing of the shortened Aar massif.

In terms of Aar massif internal deformation, previous studies^34–37^ and our field analyses^20, 38^ (Supplementary Material) indicate that Alpine deformation has a long-lasting deformation history (see below) with changing kinematics^20, 21^. In addition to previous works, we present a structural analysis along a NNW-SSE transect crossing the entire Central Aar massif (Figs 1 and 2). The analysis shows that the large-scale crustal block of the Aar massif is heavily dissected by post-collision thrusts/faults, which are expressed by steep (normal)/reverse as well as oblique-/strike slip fault zones with lateral dimensions ranging from meters up to tens of kilometers (Figs 1 and 2). Estimates on the PT conditions^37^ and exhumation history (Figs 2 and 3) indicate fault-zone nucleation at depths of at least 18–20 km^38^ suggesting large-scale structures, which dissect the entire middle to upper crust. Particularly in the granitoids (Fig. 1), some of the ductile fault zones originated at these depths as brittle faults^38^. Based on structural characterization, kinematic criteria, cross-cutting relations and absolute age constraints three different fault-zone sets can be discriminated:

(i) A NE-SW trending, steeply south dipping conjugate set of reverse and normal faults (Handegg phase^20, 38^, Supplementary Fig. 4), with dominance of S block up movements (Fig. 1a–c, Supplementary Fig. 4). Additionally, NW-SE and WNW-WSE trending oblique-slip faults with, respectively, dextral and sinistral senses of shear occur (domains a and b in Fig. 1). They are also arranged in a conjugate manner to each other and are nearly symmetrically disposed with respect the reverse/normal faults. For the central part of the transect (domain b in Fig. 1)^18^ interpreted these faults to act simultaneously during bulk horizontal NW-SE shortening. Radiometric age determination suggest an activity of these faults between 22–17 Ma^35, 36^.
(ii) The second fault-zone set (Oberaar phase^20, 38^, Supplementary Fig. 4) is most intensely developed in the south of the transect (Fig. 1 and Supplementary Fig. 4) and deviates clearly form the first set because of (a) its dominant dextral strike to oblique slip behavior, (b) the cross-cutting of the older set (see extensive discussion and appendix in ref. 20) as well as (c) radiometric ages, which start at 14–12 Ma^36^ and reach at least till 3.4 Ma^39^. Particularly the subvertical ENE-WSW trending Grimselpass shear zone^20, 38^ (Fig. 1c, Supplementary Fig. 4) is the major representative of this set, although few E-W trending large-scale dextral strike-slip faults also occur north of Grimsel Pass (see map in Fig. 1, domains b and c). As discussed in detail by ref. 20 these faults partly reactivate the older faults but additionally comprise new NW-SE and NE-SW trending strike-slip to oblique-slip faults (Fig. 1, compare strike-/oblique slip in domain c with domains a and b).
(iii) Cross-cutting of the two previous sets by NW- and SE-directed, respectively, moderately SE and SW dipping thrust-related faults planes in the north and the south, suggest a younger relative age for this third shear zone set (stereoplots in Fig. 1a and c, Supplementary Fig. 4). In the south, relatively few, very discrete and few millimeters wide fault zones occur, which accommodated limited displacements only (Fig. 1c right stereoplot). In contrast, millimeters to tens of centimeters wide NW-directed faults occur^40–42^, which show spacings in the meter to tens of meter range (Pfaffenchopf phase^42, 43^, Supplementary Fig. 4). These faults are arranged in a two kilometers wide shear-zone domain (map of Fig. 1, domain a), which accommodated an accumulated displacement of about two kilometers. From reflection seismics^27, 44^ it is recognized that also the subsurface basement-cover contact in the northern Aar massif is dissected by thrust faults (Fig. 2a), which we attribute to a similar deformation style as found at the surface. These thrust domains clearly dissect, and partly reactivate, the subvertical faults indicating a younger age^41–43^. The uppermost domain offsets zircon fission track ages of ~12 Ma^43^ suggesting an age of the NW-directed thrusting at ages younger than this time marker.

In the Aar massif, the oldest fault set (Handegg phase) evolved at peak metamorphic conditions, and is distributed along the entire N-S section (Figs 1 and 2). The fault zones occur at all dimensions with lengths ranging from the cm-scale up to the scale of tens of kilometers. Along with the metamorphic grade and background strain (Fig. 4), the spacing of the fault zones decreases towards the Grimsel Pass shear zone (Fig. 2a). Even the large faults show relatively small thicknesses of often less than a meter^20, 21^. Fault thicknesses of several meters are very rare, with the several tens of meters wide Grimsel Pass shear zone as major exception (Fig. 2a). The small fault thicknesses correlate well with very limited displacements along individual faults (<0.5–50 m), as inferred from offsets of structural markers. Although pre-Alpine folding is present in the polymetamorphic gneisses, no Alpine folds exist in the post-Variscan granitoids. Moreover, the increasing peak metamorphic grade from N to S requires substantial cumulative uplift of midcrustal rocks towards south (Supplementary Figs 2 and 3).

Based on these constraints, previous models dealing with nappe stacking, buckle folding or back thrusting can be discarded. Furthermore, the horizontal shortening and vertical flattening observed in the Central Aar massif by^17, 18^ cannot account for the observed metamorphic gradient. Instead, the orientation and distribution of the steep fault zones suggest a pervasive and complete dissection of the entire crust resulting in subvertical slices of less deformed host rocks (Figs 1, 2a and 4), similarly to a subvertically oriented card deck. Each individual fault accommodates relative small vertical displacements. Given the thousands of these slices, however, the accumulated total vertical displacement increases towards the S up to several kilometers (Supplementary Fig. 3). The most pronounced increase in the accumulated total subvertical displacement occurs in the Central part of the massif, as can be inferred from the convex shape of the peak metamorphic temperatures (Supplementary Fig. 2) as well as the steep north-dipping of the sediment-basement contact (Fig. 2a). The progression to the south into the Gotthard nappe shows a slight further increase in temperature rather than a temperature jump at the boundary to the Aar massif. Hence, the Gotthard nappe was thrusted onto the Aar massif prior to peak metamorphic conditions, i.e. the emplacement of the Gotthard was the reason for maximum burial of the Aar massif. Owing to the delay in temperature evolution, compared to pressure, the Aar Massif and Gotthard nappe therefore reached T_max_ at the same time and the same crustal level (Fig. 4c).

The first stage (Handegg phase) of substantial vertical motions of the crustal block is gradually replaced by a changing strain partitioning from the south to the north of the Aar massif. While dextral strike-slip shearing (Fig. 2a, Oberaar phase) dominates in the south, the shear domains in the north indicate a simultaneous NW-directed thrusting (Pfaffenchopf phase) exhuming the entire block further (Fig. ﻿4d). Also the subvertical flattening geometries in the Central Aar massif^17, 18, 45^ would fit perfectly with this interpretation, because NW-SE shortening in this domain (b in Fig. 1) overprints the earlier reverse faulting structures. With the rise of the Aar massif also the Gotthard nappe was further uplifted as entity with the former, i.e. without major relative vertical displacements at the boundary between the two tectonic units(Figs 4c,d and Supplementary ﻿Fig﻿. 2).

## Geodynamics: from slab rollback driven buoyancy to final compression

The presented structural framework consisting of the Aar massif, the adjacent AAT and the overlaying Gotthard nappe is responsible for the mid- to upper crustal thickening (Fig. 2a). Accordingly, the lower crust underneath these units also shows a considerable increase in thickness (Fig. 2b). What are the physical forces and processes provoking these crustal excess volumes and how do they relate to the unique subvertical deformation style described above? A reconstruction of the post-collisional deformation stages provides new insights on the associated lithosphere dynamics (Fig. 4).

Alpine subduction tectonics terminates with the breakoff of the oceanic lithospheric slab^1^ at about 34–32 Ma, where the reduced slab pulling forces resulted in decelerating European plate rollback and plate convergence rate^46^. Nevertheless, the weight of the remaining European mantle lithosphere detached from its crust promoted continued, yet slowed, rollback some 30 Ma ago^2^ (Fig. 4a). Simultaneous collisional deformation focuses mainly on the mechanical active boundaries, where at the rheological brittle-ductile transition, between the lower and middle crust, mid crustal blocks of the Gotthard nappe and AAT (i) delaminate from the bending lithosphere, (ii) experience buoyancy and uplift along a N-directed ~30° mass flow vector, and (iii) become finally accreted to the Adriatic upper plate (TALP^33^; Fig. 4a,b and e). In this in-sequence manner, the mechanical active boundary migrates northward as a function of progressive rollback and mid-crustal delamination, benefitting from the inherited crustal extensional structure of the former European passive continental margin and its pre-existing mechanical anisotropies^10, 47^. A similar delamination and buoyancy driven uplift occurs in the lower crust, though at greater depth, i.e. at stages when lower crustal rocks experience temperatures high enough to allow ductile underplating (Fig. 4c).

Despite continued lithosphere rollback, a drastic change in tectonic deformation style occurs at the moment the Aar massif starts to delaminate from its lower crust some 22–17 Ma ago (Fig. 4b,c,f), since tectonics changes from fold-and-thrust style to subvertical extrusion. At this time, the crustal-scale block of relatively intact thick and only weakly pre-structured continental crust experiences severe buoyancy forces resulting in its subvertical extrusion. As a result of the associated stress field and the initially high rock strength of the coarse-grained granitoids^21^, a dense network of steep fractures evolves (brittle precursors) along which rheology weakening in ultrafine-grained fault rocks promotes subsequent ductile faulting^21, 37, 38^. It is at this stage, when first steep shear zones start to form in the S of the Aar massif followed by a northward propagating formation of brittle/ductile fault zones. The propagation of this delamination driven midcrustal extrusion in the time interval from 20–12 Ma explains (Fig. 4c) (i) the formation of the large number of steep shear zones (probably with a slight steepening with increasing age^18^), (ii) the low displacements accommodated along each shear zone, (iii) the metamorphic gradient nowadays exposed at the surface (Supplementary Figs 2 and 3) requiring an overall dominance of S-block up movements (reverse faulting) and local S-block down movements (normal faulting), as well as (iv) the formation of the Northern steep belt with the passive rotation of the Gotthard massif in its subvertical position (Fig. 2a). Ongoing delamination in the lower crust and its buoyancy-driven piling up generates the excess of lower crust underneath the study area as manifest by the lower crustal thickening^22, 48^ (Fig. 4c). It is this post-collisional deformation with its strong steep mass flow vector, which terminates the classical Alpine collision.

Despite dominance of delamination and buoyancy processes during post-collision, a weak compressional (plate convergence) component still persisted. As documented by backthrusting in the westernmost Southern Alps^49^, this component becomes more prominent during the simultaneous late stage N-directed domainal thrusting (front) and dextral-strike slip shearing (rear) of the exhuming Aar massif (Figs 2a and 4d) at about <12 Ma. This strain partitioning promoted a further horizontal shortening and an additional tectonic uplift component of several kilometers. This late stage deformation, together with surface erosion^2^, is responsible for the shaping of the North Alpine front. Combined with the earlier vertical extrusion finally a rise of top basement from −7 km up to 5 km evolved in the time period from 20–5 Ma (vertical transport of ~12 km).

Tectonic stresses and movements still persist up to today, as documented by the ongoing seismic activity within the Alps and in the deep crust in immediate northern Alpine foreland^50, 51^. The boundary between seismically active and inactive crust follows the crust-mantle boundary down to depths of −35 km in the case of the foreland but climbs then up to −20 km underneath the Alps (yellow stippled line in Fig. 2b)^51, 52^. Interestingly, this offset correlates with the domain of thickened lower crust constructed independently (Fig. 2b). One can therefore speculate that this domain nowadays still presents a thermal anomaly where tectonic movements are accommodated in a ductile manner, while brittle processes or brittle-ductile cycles dominate in the seismically active parts. In this sense, ongoing lithosphere rollback^51^ and associated post-collisional mid- and lower crustal delamination might still be an active process as indicated by the distribution of hypocenters and recent uplift rates^53^.

This geodynamic evolution of the Aar massif with its episode of dominant subvertical tectonics might not only be representative for the exhumation of ECM in front of the European Alps but could rather reflect a general plate tectonic deformation style. We expect similar buoyancy-driven deformation styles for continent-continent collisions following classical ocean-continent subduction scenarios. Hence the entrance of the only slightly tectonically disturbed necking zones^32^ of former passive continental margins (lower plate) into the subduction zone induces (i) a fading of the subduction owing to buoyancy forces, subsequently promoting (ii) slab rollback, (iii) local temperature rises and (iii) therefore possible delamination in the lower crust. (iv) The following buoyancy-induced rise of the upper-middle crust results not only on relative movements along subvertical fault structures but also in the passive up-doming of all tectonic units which were emplaced on top before. Hence, dome-like structures at the front of young orogens might represent a hint for such geodynamic scenarios. In the case of old continent-continent collisions (e.g. Variscides, Kaledonides) one might even gain insights into the root zones (steep belts) of such vertical tectonic complexes owing to the long lasting and severe exhumation of these originally deeply buried crustal parts.

## Methods

### Tectonic sections and retro-deformation

Extensive quantitative structural analyses were carried out along the entire transect, by remote-sensing based lineament mapping, fieldwork and the reconstruction of a fault zone map, which was further processed into a 3D shear zone model^21^. The upper to midcrustal part of the tectonic NNW-SSE cross section through the Swiss Alps was constructed on the base of this 3D fault zone model, geological maps, structural field data, information available from literature and from reflection seismic data^27, 44^. While the region of primary interest, i.e. the central part of the cross section, is mainly based on own data; the upper crustal parts of the Helvetic nappes, the Penninic nappe stack and the Southern Alps was modified on the base of refs 28, 31 and 33. The upper mantle and lower crustal geometries were obtained from seismic tomography. Using an iterative approach of line-and mass-balanced retro-deformation, different orogenic stages ranging from today’s situation till the accretion of the passive European continental margin (30 Ma) were reconstructed. Here estimates on peak metamorphic conditions served as important marker points to constrain depth locations of the different tectonic units. These reconstructions served as base for the schematic summary of Fig. 4.

### Quantitative estimation of peak metamorphic conditions

Knowledge on peak metamorphic conditions (ideally associated with an age) allow to predict depths of the corresponding surface exposures at the time of maximum burial and reveal the relative changes in uplift between the different locations (Supplementary Material Figs 2 and 3). Pressure estimates for the S Aar massif (A) and the Gotthard nappe (B) derive from refs 37 and 54, respectively. Details to other data are given in Supplementary Material.

### High resolution crustal and teleseismic tomography

Seismic tomography in general terms refers to the methodology to derive 3D structural subsurface information by way of analyzing seismic waves penetrating the target volume. In addition to the controlled-source seismology originally applied in the Alpine region^22^ to study the 3D crustal structure and later the lithosphere-asthenosphere system, other seismic techniques have been extensively applied, such as, local earthquake tomography (LET), ambient noise tomography (AN), receiver functions (RF) and high-resolution teleseismic tomography (HRTET) (see ref. 14 for a summary). Note that in order to image the lithosphere-asthenosphere system with high-resolution allowing to map slab length^4^, excellent a priori knowledge about the 3D crustal structure is a prerequisite. This condition is sufficiently met for the Alpine region^55^. The presented cross section is primarily based on seismic information about the Moho topography^56^, the 3D crustal P-velocity structure^23^, and lithosphere slab geometries by HRTET^4^. Complementary seismic information constraining the locally specific geometries have been derived from CSS^44^, from AN^48, 57^ and from RF^58^.

## Electronic supplementary material

Supplementary materialPLEASE EXCHANGE THE SUPPLEMENTARY FILE WITH THE ONE ATTACHED. THE OLD ONE STILL SHOWS THE LATEST CHANGES HIGHLIGHTED IN RED.
