# Supplementary material for: Large-Scale Crustal-Block-Extrusion During Late Alpine Collision
Source: Sci Rep. 2017 Mar 24;7:413. doi: 10.1038/s41598-017-00440-0 (PMC5428471; doi:10.1038/s41598-017-00440-0)
Supplement: Supplementary file 1 — Supplementary materialPLEASE EXCHANGE THE SUPPLEMENTARY FILE WITH THE ONE ATTACHED. THE OLD ONE STILL SHOWS THE LATEST CHANGES HIGHLIGHTED IN RED. [file 41598_2017_440_MOESM1_ESM.pdf]

Supplementary Material to:

Large-Scale Crustal-Block-Extrusion During Late Alpine Collision

Marco Herwegh[1], Alfons Berger[1], Roland Baumberger[1,2], Philip Wehrens[1,2] & Edi Kissling[3]

[1] Institute of Geological Sciences, University of Bern, Baltzerstrasse 1+3, CH-3012 Bern

[2] now at Federal Office of Topography, Swiss Geological Survey, Seftigenstrasse 264, 3084 Wabern, Switzerland

[3] Institute of Geophysics, ETH Zürich, Sonneggstrasse 5, CH-8092 Zürich, Switzerland

Figures of Supplementary Material

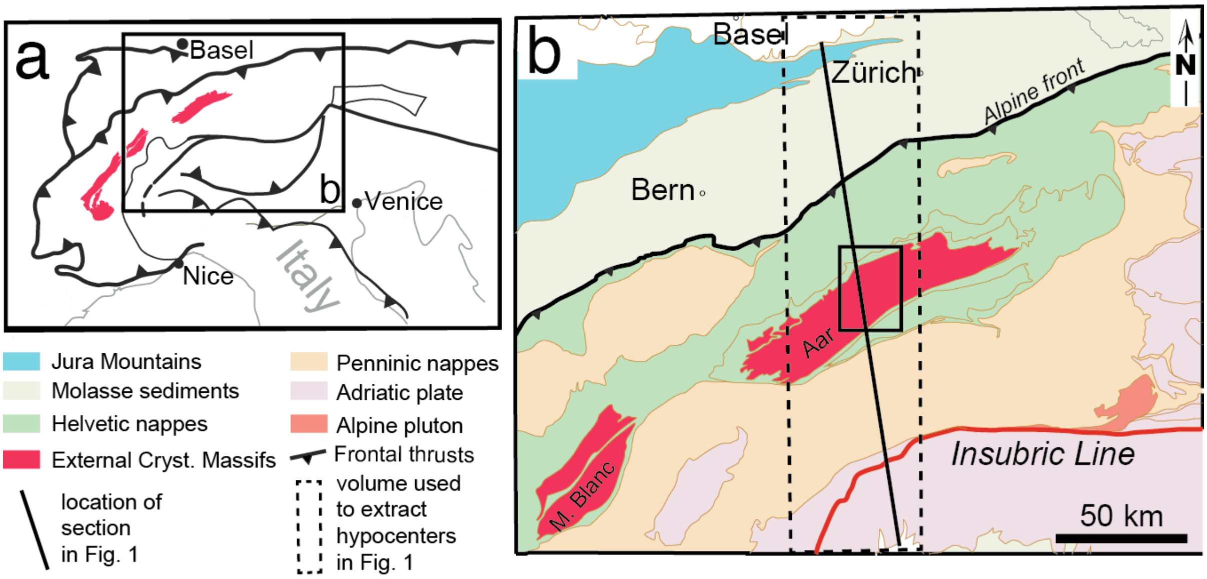

Supplementary Figure 1:
Geological map of the Central Alps: (a) overview with location of inset (b). (b) Simplified geological map of the Swiss Central Alps. Location of Figure 1 is additionally indicated (rectangular inset cutting through Aar massif). Maps were created with Adobe Illustrator CS5 (https://helpx.adobe.com/de/creative-suite/kb/cs5-product-downloads.html).

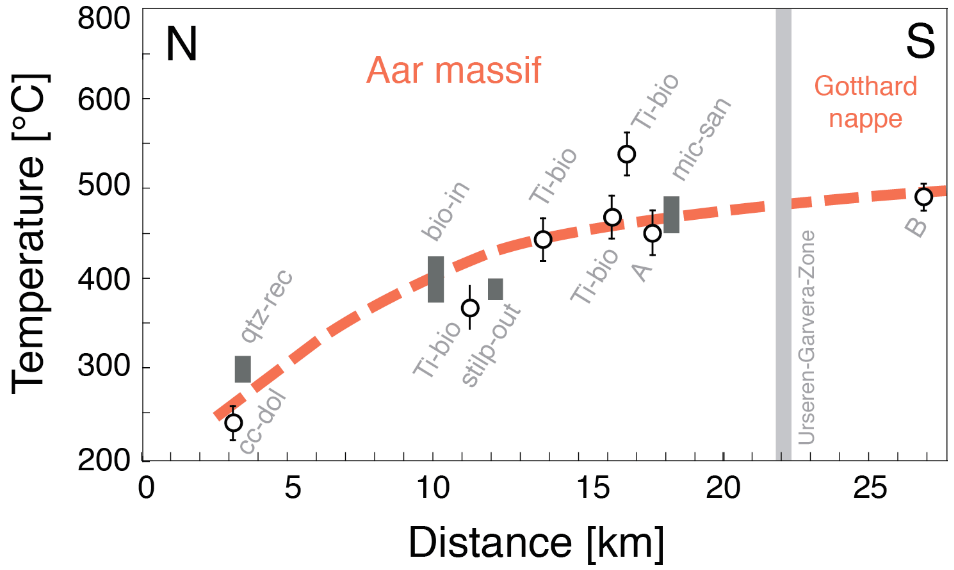

Supplementary Figure 2:

(a) Non-linear N-S increase in peak metamorphic temperatures in the N-S traverse Central Aar massif - Gotthard nappe (data in Supplementary Table 1). Cc-dol: calcite-dolomite geothermometry, qtz-rec: onset of dynamic quartz recrystallization at 280°C, Ti-bio: Titanium in biotite geothermometry, bio-in: biotite in stilp-out: stilpnomelane out mic-san microcline-sanidine transition. Data sources: the onset of dynamic recrystallization via bulging recrystallization in quartz occurs at about 280-300°C, (ii) Calcite-dolomite thermometry, (iii) biotite-in isograde, (iv) stilpnomelane-out isograde, (v) Ti in biotite thermometry and (vi) microcline-sanidine isograde. See methods of Supplementary Material for details.

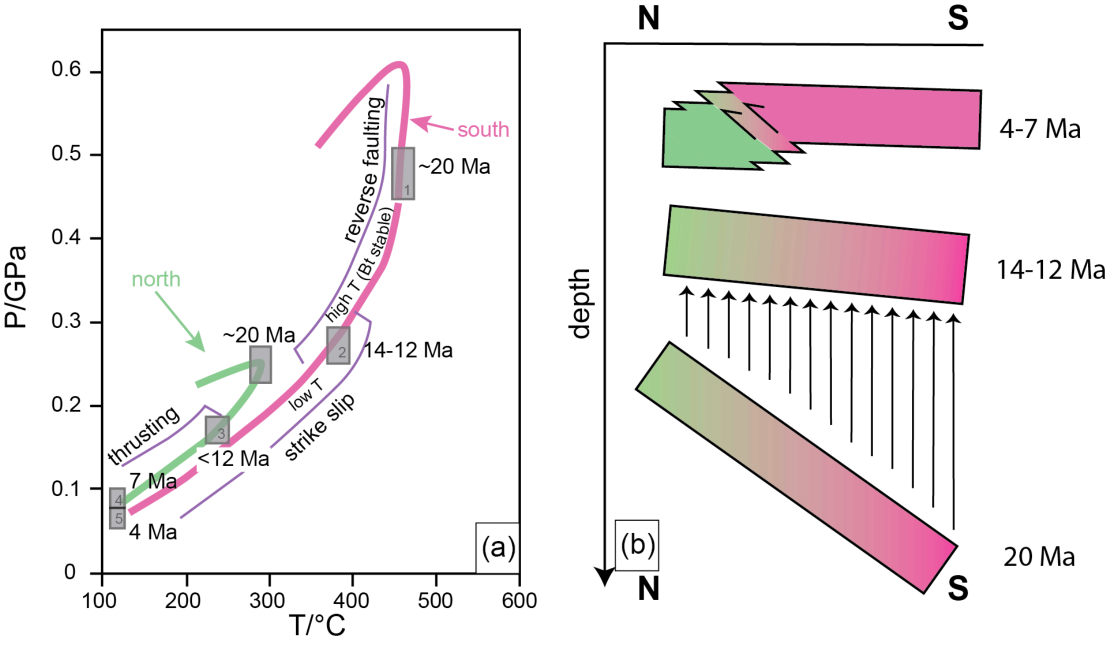

Supplementary Figure 3:
(a) P-T-t paths of the northern (green) and southern (pink) Aar massif and (b) schematic uplift of the Aar massif along the N-S Haslital transect. Note the much deeper burial in the south compared to north at 20 Ma. This depth difference is compensated by a differential vertical uplift, which increases from north to the south (see b) between 20 and 12 Ma. Afterwards, the entire Aar massif shares a similar P-T-t evolution. P-T-t paths in (a) are based on data from: (1) timing of [1] and T of [2], (2) timing of [1] and T of [3,4], (3) timing of [5,6] with temperature estimates of this study (see Supplementary Fig. 3), (4) and (5) represent apatite FT data of [6].

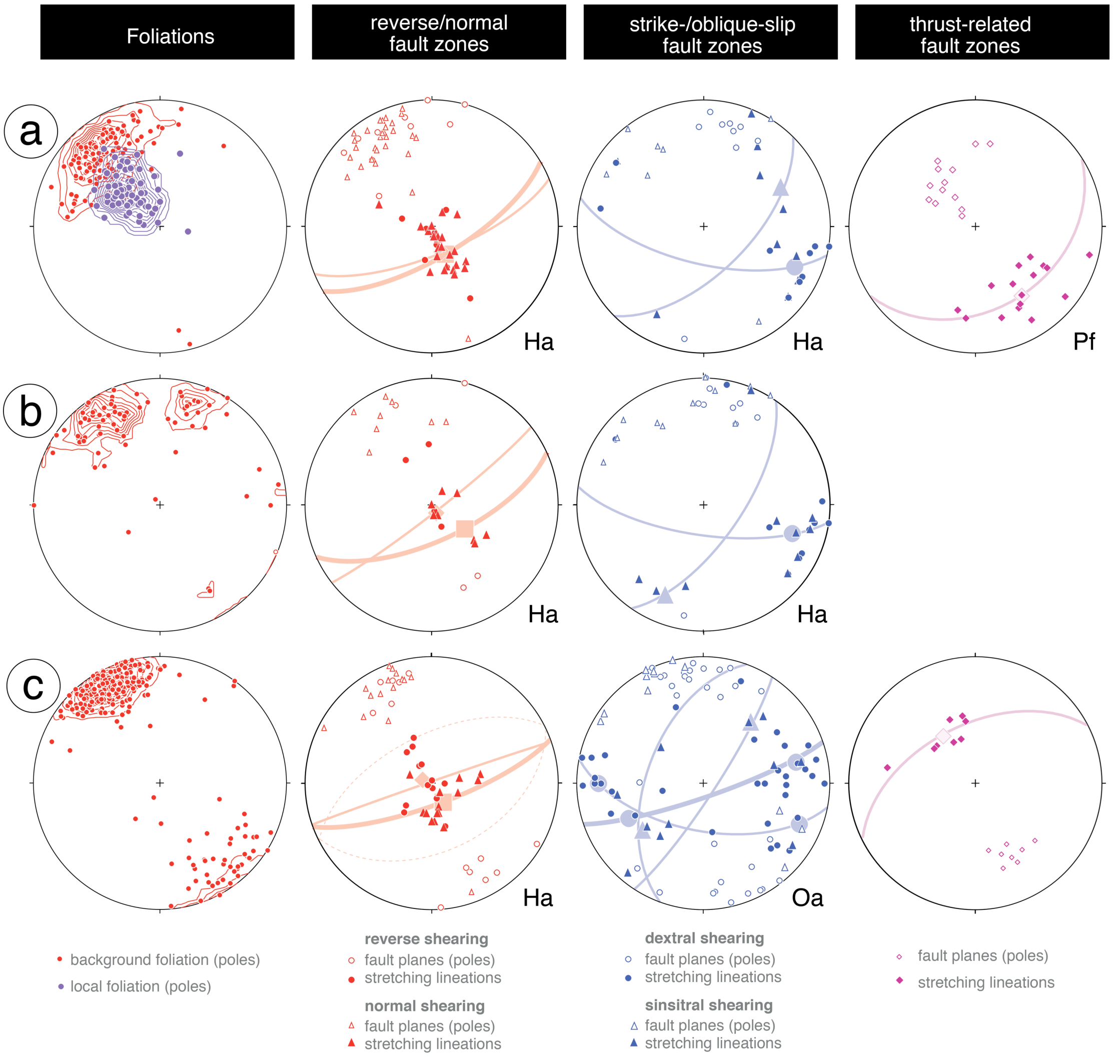

Supplementary Figure 4:
Full structural data set for the different domains (see Fig. 1). First column: foliation planes of main background strain (red, domains a-c) and local new foliation (purple, domain A), the latter being parallel to the thrust domains. Contour intervals are 2 times uniform distribution. Second column: poles of reverse/normal fault zones and stretching lineations with corresponding shear senses. Third column: poles of strike-/oblique slip zones and stretching lineations with corresponding sinistral/dextral shear senses. Fourth column: poles of thrust faults, associated stretching lineations with corresponding transport directions of the hanging wall block. Great circles show estimates of the mean fault planes and corresponding mean stretching lineations. Ha: Handegg phase, Oa: Oberaar phase, Pf: Pfaffenchopf phase structures.

Sample	Location	x-coord.	y-coord.	Method	P kbar	T (°C)	Source
GOT06-C	Gotthard Pass	~686990	~156180	Perplex	7.3	490	7
-	Grimsel	668500	159750	Trans. Micro/ Orthocl.		450-480	8
Mu320	Zinggenstock	663000	156250	Group 1 Flics	4.4	450	6
-	Rätrichs-bodensee	668640	159249	Perplex	6.2	450	2
Gr-a	GTS	667490	159300	Ti in Bio		467	this study
MP11-19	Handegg	666332	162027	Ti in Bio		440	this study
93/94	north Mittagsfluh	666075	166000	Stilpnomelan		<350	9
IN21	Pfaffenkopf	662240	171900	Cc-dol		250	this study

Supplementary Table 1:
Sample locations, approach and data used for temperature estimates.

Methods of Supplementary Material

P-T estimates
Knowledge on peak metamorphic conditions (ideally associated with an age) allow to predict depths and to reveal the relative changes in uplift between the different locations. Therefore, metamorphic data are combined form the literature and own measurements (Supplementary Table 1). We paid special attention that only peak metamorphic conditions were used and summarized in Supplementary Fig. 2. At the northern end of the massif two data sets are available: (1) the onset of dynamic recrystallization via bulging recrystallization in quartz (indicating ~280-300°C[10]); (2) carefully selected calcite/dolomite thermometry in thrust-related shear zones. The latter result in temperatures in the range of 250-280°C. The next important information toward the south is the first occurrence of newly formed Alpine biotite. This has already been described by [11], and has been supported by new Ti in biotite temperature estimates of shear zones including newly formed biotite (Supplementary Table 1). The applied Titanium in biotite geothermometry of [12] is at its limit but shows clearly the low temperature-limit of the biotite occurrence. In light of both P and T, the best investigated shear zones occur in the south of the study area and are based on thermodynamic modeling, which result in pressures of ~6 kbar and temperatures of 450°C[2] (Supplementary Table 1). This data fit with excellently with data slightly south (see[13]) and temperature estimates based on the microcline-sanidine isograde (Supplementary Table 1). All these data are plotted against distance in Supplementary Fig. 2.
The own data are based on the application of conventional geothermometers, which are applied on carefully selected microstructures. Data are measured on a JEOL JXA8200 electron microprobe using 15kV and 5-15 nA. Natural and synthetic standards are used. Calculated contents of Mg in calcite (in close neighborhood to dolomite) are transferred to temperatures using the calibration of [14]. The geothermometer of [12] was applied in biotite bearing Alpine shear zones.

Age constraints
The timing of T_{max} is strongly associated with reverse/normal faulting (Handegg phase of [15]) and has been investigated in the southern part of the study area[1,16]. The timing of the northern area results from zircon fission track data which either have either been completely reset and are offset by the thursting (Pfaffenchopf phase[3,5]) or have only been partially reset[6]. Summarizing this information two schematic P-T paths illustrate differences in P-T-t evolution between the northern and southern domains of the Haslital (Supplementary Fig. 3).

References of Supplementary Material

1. Rolland, Y., Cox, S.F. & Corsini, M. Constraining deformation stages in brittle-ductile shear zones from combined field mapping and 40Ar/39Ar dating: The structural evolution of the Grimsel Pass area (Aar Massif, Swiss Alps). *Journal of Structural Geology* **31**, 1377-1394 (2009).
2. Goncalves, P., Oliot, E., Marquer, D. & Connolly, J.A.D. Role of chemical processes on shear zone formation: an example from the Grimsel metagranodiorite (Aar massif, Central Alps). *Journal of Metamorphic Geology* **30**(7), 703-722, doi:10.1111/j.1525-1314.2012.00991.x (2012).
3. Wehrens, P. Structural evolution in the Aar Massif (Haslital transect): Implications for mid-crustal deformation. *PhD thesis University Bern* (2015).
4. Mullis, J. PTt path of quartz formation in extensional veins of the Central Alps: *Schweizerische Mineralogische und Petrographische Mitteilungen* **76**(2), 159-164 (1996).
5. Herwegh, M., Mock, S., Wehrens, P., Baumberger, R., Berger, A., Wangenheim, C., Glotzbach, C., and Kissling, E., The Front of the Aar Massif: A Crustal-Scale Ramp Anticline? *in Proceedings EGU General Assembly Conference Abstracts 2015* ***17,*** 11769 (2015).
6. Michalski, I. & Soom, M. The Alpine thermo-tectonic evolution of the Aar and Gotthard massifs, Central Switzerland: fission track ages on zircon and apatite and K–Ar mica ages. *Schweizerische Mineralogische Petrographische Mitteilungen* **70**, 373-387 (1990).
7. Oliot, E., Goncalves, P. & Marquer, D. Role of plagioclase and reaction softening in a metagranite shear zone at mid-crustal conditions (Gotthard Massif, Swiss Central Alps). *Journal of Metamorphic Geology* **28/8**, 849-871 (2010).
8. Bambauer, H. U., Bernotat, W., Breit, U. & Kroll, H. Perthitic alkali feldspar as indicator mineral in the Central Swiss Alps. Dip and extension of the surface of the microcline/sanidine transition isograd. *European Journal of Mineralogy* **17**(1) 69-80, doi:10.1127/0935-1221/2005/0017-0069 (2005).
9. Niggli, E. & Niggli, C. Karten der Verbreitung einiger Mineralien der alpidischen Metamorphose in den Schweizer Alpen (Stilpnomelan, Alkali-Amphibol, Chloritoid, Staurolith, Disthen, Sillimanit). *Eclogae Geologicae Helvetiae* **58**, 335-368 (1965).
10. Stipp, M., Stünitz, H., R. Heilbronner R. & Schmid, S.M. The eastern Tonale fault zone: a 'natural laboratory' for crystal plastic deformation of quartz over a temperature range from 250 to 700°C. *J. Struct. Geol.* **24**,1861-1884 (2002).
11. Steck, A. & G. Burri, G. Chemismus und Paragenesen von Granaten aus Granitgneisen der Grünschiefer- und Amphibolitfazies der Zentralalpen. *Schweiz Mineral Petrogr Mitt.* **51**, 534-538 (1971).
12. Henry, D. J., Guidotti, C.V. & Thomson, J.A. The Ti-saturation surface for low-to-medium pressure metapelitic biotites: Implications for geothermometry and Ti-substitution mechanisms. *American Mineralogist* **90/2-3**, 316-328 (2005).
13. Diamond, L. W. & Tarantola, A. Interpretation of fluid inclusions in quartz deformed by weak ductile shearing: Reconstruction of differential stress magnitudes and pre-deformation fluid properties: *Earth and Planetary Science Letters* **417**, 107-119 (2015).
14. Anovitz, L. M. & Essene, E. J. Phase equilibria in the system $CaCO_3$-$MgCO_3$-$FeCO_3$. *Journal of Petrology* **28**, 389-414 (1987).
15. Wehrens P., Baumberger, R., Berger, A., Herwegh, M. How is strain localized in a meta-granitoid, mid-crustal basement section? Spatial distribution of deformation in the Aar massif (Switzerland). *Journal of Structural Geology*, *Journal of Structural Geology* **94**, 47-67 (2017).

16. Challandes, N., Marquer, D., Villa, I. P-T-t modelling, fluid circulation, and 39Ar-40Ar and Rb-Sr mica ages in the Aar Massif shear zones (Swiss Alps). *Swiss Journal of Geosciences* **101**, 269-288 (2008).
